# Feed additives and enrichment materials to reduce chicken stress, maximize productivity, and improve welfare

**DOI:** 10.14202/vetworld.2024.2044-2052

**Published:** 2024-09-13

**Authors:** Karim El-Sabrout, Stefano Landolfi, Francesca Ciani

**Affiliations:** 1 Department of Poultry Production, Faculty of Agriculture, Alexandria University, Alexandria 21545, Egypt; 2 Department of Veterinary Medicine, University of Bari Aldo Moro, Apulia 70010, Italy; 3 Department of Veterinary Medicine and Animal Production, University of Naples Federico II, Naples 80138, Italy

**Keywords:** antioxidants, behavior, environmental enrichment, gut health, product quality, stress, welfare

## Abstract

Environmental stress poses serious threats to animal welfare and production, particularly in poultry, which are susceptible to such stress. It can increase susceptibility to diseases and infections, reduce growth rates and reproductive performance, and increase behavioral issues. Environmental stress caused by conventional housing conditions can negatively affect well-being and productivity. High temperature, overcrowding, poor ventilation, insufficient lighting, and wire cages are some of the most prominent stressors in conventional housing systems. To address environmental stress in chicken farms, some strategies and tools, such as using anti-stress feed additives and enriching cages, can help improve bird behavioral activities and welfare. Breeders can improve overall bird performance by implementing these strategies and creating a more enriched and comfortable environment. Thus, this review discusses the importance of using different feed additives and environmental enrichment materials to reduce stress in chicken farms (broiler and layer) and improve bird productivity and well-being.

## Introduction

Due to minimum initial investment, small production area, high fecundity, and ability to feed fibrous diets, poultry farms are among the sustainable agricultural industries that provide high-quality animal protein [1]. Recently, consumers have expressed great interest in improving animal welfare and product quality [2]. Prioritizing animal welfare aligns with ethical principles and enhances product quality, leading to increased consumer satisfaction and long-term benefits for both animals and consumers.

Feed additives and enrichment materials play crucial roles in reducing stress and improving the overall welfare of chickens during poultry production. Environmental stress in poultry is a significant concern in the poultry industry because it can harm birds’ health and well-being [3, 4]. Environmental stressors include temperature fluctuations, poor air quality, overcrowding, and inadequate lighting [5–10]. These stressors can increase disease susceptibility, decrease egg production, and even lead to death in extreme cases. It is essential for poultry producers to be aware of these stressors and to take appropriate measures to mitigate their effects to ensure the welfare of their birds and maintain productivity. However, stress must be addressed through several methods, most notably dietary additive supplementation and management (including facilities). These strategies can decrease stress, improve animal comfort, health and well-being, and maintain animal performance.

By incorporating feed additives, such as cinnamon, turmeric, vitamins (C and E), minerals (zinc and manganese), and enzymes, such as nicotinamide adenine dinucleotide phosphate oxidase, into chickens’ diets, producers can help maintain a healthy gut microbiota and optimize nutrient absorption, leading to improved immune function and overall performance [4, 11]. In addition, enrichment materials can also be used to reduce stress and improve chicken welfare in a production setting. Enrichment materials such as perches, pecking objects, mirrors, and dust baths can stimulate birds’ natural behaviors and reduce aggressive behaviors and cannibalism in flock environments [12, 13]. Providing chickens with access to enrichment materials improves their welfare and promotes natural behavior, leading to healthier and more contented birds [13, 14].

In general, incorporating feed additives and enrichment materials into the management of chicken production can significantly benefit the health and welfare of the birds and maximize profitability [2, 13, 15, 16]. Producers can improve productivity, reduce disease incidence, and create a more sustainable and affordable production system by prioritizing chickens’ mental and physical well-being. Chicken producers should consider using these tools in their management practices to ensure the highest animal welfare and efficiency standards. Therefore, this review highlights the importance of using different feed additives and enrichment materials, either individually or in combination, to reduce chicken stress and improve performance and welfare.

## Feed Additives to Reduce Chicken Stress, Maximize Productivity, and Improve Welfare

This section discusses the most practical food additives used during seasons of environmental stress, particularly heat stress, for chickens raised in tropical and Middle Eastern countries. One of the key benefits of using feed additives to reduce chicken stress is their ability to modulate the bird’s stress response. Shi *et al*. [17] observed that heat stress, the most significant environmental stressor in chickens, causes profound changes in the gut microbiota composition. Heat stress significantly affects species abundance and diversity. It can affect growth performance by altering the gastrointestinal microbiota, leading to increased permeability and immunological and metabolic dysfunction. Thus, when evaluating nutritional additives in monogastric animals like chickens, key parameters such as gut health, antioxidant-related factors, growth promoters, reproductive stimulators, hematological and biochemical indicators, immune status, and carcass/meat quality should be considered. In recent years, the complex interactions among intestinal bacteria, epithelial barriers, and immune cells in the gastrointestinal system have attracted great interest [18, 19]. Gut health has a significant impact on a bird’s overall performance. Certain compounds, such as antioxidants and probiotics, have been shown to lower stress hormone levels in poultry, resulting in a calmer and more relaxed flock [20, 21]. In addition, these additives can improve the gut health of birds, which is closely linked to their overall stress levels [11, 21]. By maintaining a healthy gastrointestinal microbiome, chickens can better cope with environmental stresses and maintain resilience despite challenges.

Furthermore, feed additives can enhance the immune function of chickens, providing them with greater protection against diseases and infections [22]. Stress weakens the immune system of birds, increasing their susceptibility to illness. By fortifying their diets with immune-boosting ingredients, such as vitamins and minerals, poultry producers can help their birds maintain a strong defense against pathogens [23]. This benefits individual birds and reduces the risk of disease outbreaks within the flock, leading to better overall productivity and profitability for producers. The antioxidant potential of vitamins and micro-minerals is more efficient in combination with heat stress in chicken nutrition [24, 25]. Botanical extracts may reduce the adverse effects of heat stress on antioxidant enzyme activity because of their antioxidant components [20, 26]. Furthermore, betaine can reduce heat production in birds at high ambient temperatures [24]. Thus, it is recommended that chicken should be fed under tropical conditions. Righi *et al*. [27] observed that using plant feed additives as antioxidants in poultry diets is an effective strategy for combating oxidative stress under various conditions, frequently with beneficial effects on poultry productivity and efficiency.

Vitamins such as E, C, and A can react with free radicals, lowering their levels and lipid peroxidation in birds [25, 27]. However, microminerals such as Zn and Se are not directly capable of preventing or reducing reactive oxygen species formation, but they are essential cofactors of enzymes that react with free radicals [24]. Vitamins E and C supplementation improve the antioxidant properties of critical enzymes, such as catalase (CAT), glutathione reductase, glutathione peroxidase, and superoxide dismutase (SOD) [24, 25]. Zn and Se also enhance antioxidant enzyme activity (e.g., glutathione reductase and glutathione peroxidase) [24]. Mohamed *et al*. [28] observed that applying feed restriction for 3 h or dietary supplementation with 200 mg Vitamin C + 200 mg Vitamin E + 1.5 mg Cr + 100 mg Zn/kg for stressed broilers under summer conditions could significantly alleviate heat impacts by improving performance parameters, blood biochemical indices, and carcass quality. Two methods have been employed to mitigate the effects of heat stress on broiler chicks using dietary management strategies: (1) Feed restriction and (2) feed additives [28, 29].

The major effects of alternative feed additives include enhancing digestion, increasing the absorbability of nutrients, improving nutrient availability, enhancing antioxidant activity, boosting immunity and antimicrobial effects, improving intestinal health by enhancing gut integrity, modulating host gut microflora, and improving intestinal barrier function [30]. Moreover, feed additives increase body and carcass weight, enhance feed conversion ratios, and improve the gut health of broilers [4, 30]. Enzymes, which are an important feed additive in chicken diets, act as catalysts, aiding digestion and nutrient use that would otherwise be wasted by the body. They decrease the anti-nutrient effects of non-starch polysaccharides in plant cell wall components found in cereal grains and oil seeds [31].

Previous studies by Abd El-Aziz *et al*. [19] and Lan *et al*. [32] have demonstrated that sodium butyrate enhances the intestinal defense system, lowers oxidative stress, exhibits immunomodulatory and anti-inflammatory properties, and supports intestinal integrity and microbial balance. Furthermore, Yang *et al*. [33] demonstrated that the intestinal microbiota of broiler chicks is positively affected by butyrin, thereby improving chicken health. To shed light on the effects of feeding broiler chickens, a diet enhanced with vitamin D_3_ (VD_3_) and sodium butyrate on their meat quality, oxidative stability, and nutritional value. Gao *et al*. [34] reported an increase in 2,2-diphenyl-1-picrylhydrazyl (DPPH) and 2,2′-azinobis-3-ethylbenzothiazoline-6-sulfonic acid (ABTS) levels in chicken meat, suggesting an enhancement in antioxidant ability. Significantly, the concurrent presence of elevated VD_3_ levels and sodium butyrate increased the concentration of polyunsaturated fatty acids (PUFAs) in chicken meat. Thus, incorporating sodium butyrate into the diet greatly improved chicken meat quality. This improvement may be mainly attributable to increased antioxidant capacity and improved physical properties [34]. Xiao *et al*. [35] reported a considerable increase in the concentrations of immunoglobulin A in the blood of both broiler breeders and their offspring following maternal supplementation with sodium butyrate. In addition, it caused the progeny’s immunoglobulin G levels to rise noticeably [35]. The results of this study highlight sodium butyrate’s ability to strengthen broiler breeders’ immune systems and increase their offspring’s antioxidant capacity.

Furthermore, Lan *et al*. [32] investigated how resistance to heat-related oxidative stress in broiler hens is affected by three dietary levels of sodium butyrate (300, 600, and 1200 mg/kg). When sodium butyrate was administered to the breast muscle, the levels of SOD, glutathione peroxidase, and CAT increased significantly, whereas malondialdehyde (MDA) levels showed a tendency toward reduction. These findings demonstrate that sodium butyrate is a potential dietary supplement for broilers in hot climates because it boosts their antioxidant capacity, which is useful for chicken health. Furthermore, Deng *et al*. [36] studied how adding xylo-oligosaccharides and coated sodium butyrate to the feed of broiler chickens affected their antioxidant and anti-inflammatory abilities. Consequently, total antioxidant capacity, SOD, interleukin-10, and transforming growth factor-beta levels were increased by adding coated sodium butyrate and xylo-oligosaccharides, either alone or in combination.

Curcuminoids are key antioxidants in turmeric [37]. Curcumin (the active extract of turmeric) increases the levels of major detoxifying enzymes known as glutathione-S-transferase. It has an intrinsic ability to absorb free radicals, especially oxygen species [2, 38]. Ahmadi [39] studied the effects of 0.3 and 0.6 g/kg turmeric powder supplements administered to birds under heat stress. The superoxide radicals were neutralized, and there was an increase in the activity of SOD and CAT and a decrease in MDA in broilers [39]. Curcumin has a considerable ability to scavenge superoxide hydrogen peroxide radicals and nitric oxide from activated macrophages, decrease lipid peroxidation, reduce iron complex, and increase antioxidant status, particularly through the activity of SOD, which may be caused by enhanced SOD gene expression in hens fed turmeric [40, 41]. Liu *et al*. [42] reported that dietary supplementation with 200 mg/kg curcumin significantly improved the egg quality of quails in the late laying period by improving antioxidant activity, lipid metabolism, and maintaining the intestinal microbial community. According to Sugiharto [43], turmeric reduces the harmful effects of heat stress in broiler chickens in terms of productivity, physiology, immunology, and antioxidant status. Therefore, adding turmeric (curcumin) to chicken diets has several benefits, particularly under stressful conditions, because it can reduce stress in birds. However, cinnamon oil also has antioxidant and hypocholesterolemic properties, improving broiler meat quality [4, 44]. Ali *et al*. [45] found that cinnamon supplementation in poultry diets alleviates stress responses by suppressing the nuclear factor kappa B pathway and increasing anti-inflammatory cytokine expression. It can be used as a feed additive in chicken farms to improve health, food safety, and economic outcomes. According to Khan *et al*. [46], turmeric powder (1%) and cinnamon powder (1%) have a positive impact on liver function, growth performance, antioxidant status, meat quality, and lactate dehydrogenase activity. Birds that received powdered cinnamon and turmeric had increased blood SOD, CAT, and glutathione peroxidase activities, whereas serum MDA and lactate dehydrogenase activities decreased. Furthermore, alkaline phosphatase, alanine aminotransferase, and aspartate aminotransferase activities were reduced by adding cinnamon and turmeric. Supplementation with cinnamon and turmeric increased breast and thigh meat PUFAs, monounsaturated fatty acids, oleic acid, linoleic acid, and water-holding capacity while decreasing saturated fatty acid content [46].

Dietary additives may be useful in mitigating the negative effects of heat stress, as well as improving growth and laying performance, meat and egg quality, antioxidant status, and immunological response [47]. However, more research is needed to investigate the combined effect of some of the aforementioned nutritional strategies, either alone or in conjunction with management tools, to ameliorate the negative impacts of heat stress and to examine their effectiveness and cost-benefit in the poultry industry.

## Enrichment Materials to Reduce Chicken Stress, Maximize Productivity, and Improve Animal Welfare

Environmental enrichment can include providing chickens with opportunities for social interactions, access to a variety of perches, nesting boxes, and other structures, and access to different textures, such as straw or wood chips, for scratching and foraging [15, 48]. Environmental changes threaten animal welfare and production, particularly for poultry, which are sensitive to such changes. For example, changes in temperature (extreme heat or cold) can cause significant stress to vital biological functions and the body [49]. High temperatures, in particular, can lead to heat stress, resulting in reduced feed intake, decreased egg production, and even mortality [50, 51]. Environmental stress influences not only the behavioral activities of animals but also their physiological responses, including activation of the nervous and hormonal systems, which have the greatest impact on animal health and productivity [8, 52, 53].

Furthermore, stress has a profound impact on the genetic structure of birds, leading to harmful effects on their DNA integrity [54, 55]. When animals encounter stressors resulting from environmental factors or social interactions, their bodies initiate a cascade of physiological responses to cope with the perceived threat [56, 57]. Behavioral responses and the release of stress hormones (such as cortisol and adrenaline) trigger a series of adaptive changes that enhance survival [21]. However, producers and breeders must provide adequate ventilation, cooling systems, and shade to help poultry regulate their body temperatures and minimize the risk of environmental stress.

Previous studies [58, 59] in chickens have demonstrated that stress may cause changes in gene expression, altering critical biological functions, such as metabolism, reproduction, and immune response. In addition, stress-induced changes in DNA methylation patterns are linked to increased susceptibility to diseases and reduced overall fitness of these animals. One of the most alarming consequences of chronic stress is DNA damage in animals [56, 59]. Oxidative stress is a byproduct of prolonged exposure to stress hormones and can cause DNA strand breaks and mutations, thereby compromising the stability of the genome [60]. DNA damage in chickens affects aging and increases the risk of developing genetic disorders and hereditary diseases.

Conventional housing systems are a major source of stress for birds. Several studies and investigations have been conducted to address these issues and provide solutions based on basic practical strategies. From the previous studies by Son *et al*. [13], Belz *et al*. [61], and Elsayed *et al*. [62], environmental enrichment aims to keep animals entertained and active while preventing behavioral issues and maintaining their mental health. Belz *et al*. [61] defined environmental enrichment as the use of numerous objects to improve the living quality and normal behavior expression of animals in cages or confined spaces. Previous studies [63, 64] have shown visual, sound, and rubber object enrichment strategies for poultry houses. These strategies have significant potential for improving animal health, productivity, welfare, and farm management.

Although the evaluation of the affective experiences of birds is a new concept, there are methods for assessing the efficacy of enrichment from a bird’s perspective. The relationship between bird behavior and health must be considered when developing environmentally enriching methods for poultry, especially broiler chickens. Genetics, age, stocking density, temperature, and lighting affect enrichment efficacy. Several enrichment materials can provide direct and indirect benefits for other aspects of bird health and welfare, in addition to behavioral improvement. For example, perches, platforms, balls, and mirrors may help bone and muscle growth and reduce contact dermatitis. In addition to their nutritional importance, live insects (e.g., worms) can encourage locomotory and foraging behaviors, which can improve walking abilities [3, 65]. According to Jacobs *et al*. [3], creating a complex environment and combining various enrichments can improve bone strength, walking ability, and the risk of scratches and mortality.

Enrichment materials were selected based on their perceived low hygiene risk and simplicity in removal during production cycles. De Jong and Gunnink [64] investigated the effect of supplying broiler chickens with a combination of wood shaving bales (1.5 bales/1000 chicks), round metal perches (2.7 m/1000 chicks), and metal chains (1/1000 chicks) as pecking items. They discovered enriched birds that engaged in much more walking, exploring, and foraging than the control group. They also proposed conducting additional research on combinations of environmental enrichments to improve the activity of fast-growing broiler chickens and other welfare aspects, such as their walking ability. Similarly, Bailie and O’Connell [66] reported that environmental enrichment improves the psychological well-being of birds. It reduces fear in several species of poultry. In addition, the treated birds had fewer hock burns, toe damage, and footpad dermatitis. The improved general health of the birds was probably caused by their early and quick response to enrichment methods and acclimatization to the bedding material.

According to Yildirim and Taskin [67], environmental enrichments, such as mirrors, balls, dust, and perch, improve the welfare of birds while having no negative effects on growth performance or lymphoid organ weight. Likewise, Zahoor *et al*. [15] found that broiler chickens raised under various environmental enrichments were more active and exhibited maintenance activities (e.g., dust bathing, wing stretching, and preening). Environmental enrichment can also affect certain blood biochemical parameters and productive traits. Son *et al*. [13] found that providing pumice stone and alfalfa hay as enrichment materials to laying hens in the aviary system decreased blood creatinine levels and significantly lowered corticosterone levels. They also reported that these materials improve egg production, alleviate stress, and maintain health. According to Zahoor *et al*. [15], chickens reared with red balls had the lowest cholesterol, glucose, and total protein levels compared with other studied groups. Chicks reared with green balls exhibited the highest body weight at slaughter, dressed weight, and carcass yield. Zahoor *et al*. [15] concluded that the addition of environmental enrichment tools (visual, structural, and plastic) motivated birds to perform physical activities while improving broiler chicken behavior, growth, and carcass quality. Elsayed *et al*. [68] indicated that enriching the rabbit environment with inexpensive materials and tools, like plastic-colored balls and mirrors, improves rabbit production, carcass quality, behavior, and welfare. It also reduces the fear response of rabbits and improves their health and immunity.

Modern broilers are typically grown under barren conditions in enclosed housing. Several environmental enrichment components, such as elevated platforms and perches, have been proposed for these barns to improve bird welfare [12, 13, 15]. Broiler birds strongly prefer elevated platforms over combined structures or simple perches. By providing incentives to climb elevated areas, for example, environmental enrichment can target issues associated with inactivity while giving birds greater flexibility to display a larger range of targeted behaviors [69]. On the other hand, mirrors add an additional dimension to the environment and increase the space in which animals are located. Hillemacher *et al*. [70] reported that birds could clearly distinguish between their reflections and the sights of others. Zahoor *et al*. [15] reported that birds raised with mirrors exhibited considerably more prominent maintenance activities, such as preening, dust bathing, and wing scratching, than the control group. Weight gain and feed intake in birds reared with mirrors were also improved on days 21 and 35, respectively. Furthermore, mirror enrichment reduces the physiological stress caused by dense stockings [71].

## Future Perspectives

**Figure-1:**
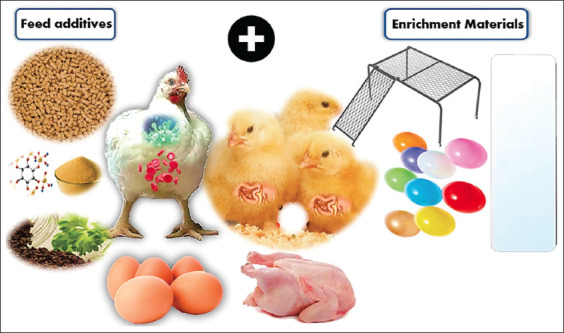
Use of appropriate feed additives and effective enrichment materials combined to improve chicken health and produce high-quality products.

As global demands for poultry production and welfare increase, precision poultry farming strategies have become increasingly vital to solving existing animal welfare challenges and production efficiencies. A single strategy cannot mitigate the effects of environmental stress on chickens. Thus, using appropriate feed additives with effective enrichment materials can help reduce environmental stress in chicken farms (Table-1) [12, 13, 15, 19, 24, 25, 28, 39, 42, 46, 69, 72]. Applying these primary potential mitigation strategies together, particularly under stressful conditions, allows birds to better express and maximize their natural behaviors (Figure-1). In agreement, Wasti *et al*. [73] reported that proper management and nutritional management should be considered to mitigate environmental stress in birds.

**Table-1:** Potential feed additives and enrichment materials to reduce environmental stress and improve productivity in chicken farms.

Mitigation strategies	Outcome/Mechanism	Reference
Adding straw bales, step platforms, and laser projectors	Increase locomotion, reduce fear expression, and improve welfare indicators of broiler chickens.	[12]
Pumice stone and alfalfa hay provision to laying hens	Improve egg production, health, and alleviates stress.	[13]
Mirrors and colored balls enrichment in broiler houses	Increases body weight at slaughter and carcass yield. It also motivates birds’ physical activities and improves their welfare.	[15]
Sodium butyrate (300–500 g/t) inclusion	Improves gut wall tissue development, antioxidant capacity, and immune response, resulting in improved feed conversion ratio, body weight, and product quality.	[19]
Vitamin E and C (200–300 mg/kg diet) supplementation	Protects against oxidative damage and acts as an antioxidant, improving overall performance, reducing metabolic heat generation, and enhancing immunological responses.	[24]
Zn (30–60 mg/kg diet) and Se (0.5–1 mg/kg diet) supplementation	Help maintain a healthy gastrointestinal microbiota and optimize nutrient absorption, leading to improved immune function and overall performance.	[25]
Feed restriction (3 h/d) and Vitamin C supplementation (200 mg/kg diet)	Alleviate heat impacts by improving performance parameters, blood biochemical indexes, and carcass quality.	[28]
Turmeric or cinnamon powder (1%) supplement	Improve antioxidant status, blood biochemical index, immunity, and carcass quality.	[46]
Curcumin (50–200 mg/kg diet) administration	Enhances bird health and meat/egg quality by reducing lipid peroxidation and increasing the antioxidant capacity.	[39, 42, 72]
Elevated platforms combined with perches	Maintains growth performance and improves welfare.	[69]

El-Baz and Khidr [30] explained the importance of feed additives in the context of marginal environments, which present several challenges for chicken feeding. In addition, feed additives will be useful tools to address other environmental challenges under current conditions. Herbal essential oils, for example, can improve gut health and immune function, whereas enrichment materials, such as mirrors, can enhance behavioral and psychological well-being in chickens [4, 15, 62, 74]. It is well known that heat stress is a major environmental stressor in the poultry industry, resulting in substantial economic losses [73]. The most recent strategy to reduce heat stress in poultry is early perinatal programming using the *in ovo* technology [75]. As such, this strategy is expected to be more efficient and cost-effective in mitigating the effects of heat stress on poultry and improving the performance and health of birds. The *in ovo* supplementation of bioactive substances has several advantages over other strategies to mitigate heat stress in chickens. These strategies include improving immune functions, increasing nutrient absorption, and promoting antioxidant activities while reducing rectal temperature and subsequently generating thermotolerance in chickens [76]. Furthermore, the administration of bioactive substances to chick embryos can establish lifelong phenotypes, including superior performance, immunity, and a healthy gut microbiome in birds [77].

In addition to environmental enrichment applications, advanced technologies are important for detecting abnormal behaviors, physiological issues, and illnesses in chicken farms. Sensors, for example, play a crucial role in collecting data on daily animal behavioral activities and physiological parameters [78]. By tracking these metrics, breeders can gain valuable insights into the welfare of their animals and choose the best environmental enrichment approach, as well as implement targeted interventions to address any issues. Moreover, monitoring systems provide real-time data on several environmental parameters, such as temperature, humidity, and harmful gases (such as ammonia) [79–81]. Breeders can use artificial intelligence to enhance efficiency, minimize resource wastage, and improve overall farm performance.

In recent years, the use of cybernetic systems to promote modern poultry farming has become increasingly prevalent. These advanced technologies have revolutionized how poultry farmers operate by providing them with real-time data and analytics to optimize their production processes. Farmers can closely monitor their birds’ health, behavior, and performance by integrating sensors, cameras, and automated systems into poultry farms, leading to improved efficiency and productivity [82, 83]. These cybernetic systems have transformed the poultry farming industry by streamlining operations and reducing manual labor, ultimately leading to increased profitability for farmers.

The intensive nature of chicken farming practices can lead to environmental challenges and impact animal welfare; therefore, the integration of appropriate feed additives and suitable enriching materials with sensors and monitoring systems is crucial for improving the environmental conditions of chicken farms. Producers and breeders can exploit these practices and technologies to improve bird welfare, optimize farm productivity, and mitigate environmental impacts. These strategies could be part of a farm management system for the early detection of potential problems and the implementation of proactive measures to ensure that birds are maintained under optimal conditions. Furthermore, it is advisable to conduct further research and investigation to explore novel mitigation strategies in terms of nutritional manipulation and management practices to ensure sustainable well-being and production.

## Conclusion

Chicken meat is a primary animal protein source in several countries. The chicken industry has recently faced significant husbandry challenges from various environmental stressors. Feed additives and enrichment materials are essential for optimizing chicken health, welfare, and productivity in intensive production systems. These two strategies are crucial for reducing environmental stress and improving birds’ overall well-being. By applying these strategies, poultry producers and breeders can create a more enriched and comfortable environment and healthier and more resilient flock, leading to improved growth rates, higher quality meat and eggs, and, ultimately, more sustainable poultry production.

## Data Availability

The supplementary data can be available from the corresponding author upon request.

## Authors’ Contributions

KES: Conceptualization, supervision, and visualization of the study. KES and FC.: Data collection and curation. KES, SL, and FC: Wrote and revised the manuscript. All authors have read, reviewed, and approved the final manuscript.
